# Explaining the slow transition of child-appropriate dosage formulations from the global to national level in the context of Uganda: a qualitative study

**DOI:** 10.1186/s40545-015-0039-1

**Published:** 2015-07-15

**Authors:** Xavier Nsabagasani, Ebba Hansen, Anthony Mbonye, Freddie Ssengooba, Herbert Muyinda, James Mugisha, Jasper Ogwal-Okeng

**Affiliations:** Child Health and Development Center, College of Health Sciences, Makerere University, P.O. Box 6717, Kampala, Uganda; Section for Social and Clinical Pharmacy, Department of Pharmacy, Faculty of Health and Medical Sciences University of Copenhagen, Copenhagen, Denmark; Ministry of Health Uganda and Department of Health, Uganda Christian University, Kampala, Uganda; School of Public Health, College of Health Sciences, Makerere University, Kampala, Uganda; Departments of Pharmacology and Therapeutics, Gulu University, Gulu, Uganda

**Keywords:** Children, Child-appropriate dosage formulations, Stakeholders, Policy transfer, Child size medicines and Uganda

## Abstract

**Background:**

In 2007, the Sixtieth World Health Assembly (WHA) passed a resolution entitled “Better medicines for children” and subsequently the World Health Organization (WHO) recommended the inclusion of child-appropriate dosage formulations in the essential medicines lists of member countries. However, child-appropriate dosage formulations are not highlighted in the Essential Medicines and Health Supplies List of Uganda (EMHSLU) 2012 and they are still limited in availability in public health facilities. Several stakeholders influenced the status of child-appropriate dosage formulations in the EMHSLU 2012.

**Objective:**

To explore stakeholders’ views about the relevance of the globally recommended child-appropriate dosage formulations in the context of Uganda.

**Methods:**

The findings derive from thirty three in-depth interviews with stakeholder representatives and the results of a follow up validation meeting where preliminary findings were shared with stakeholders. Policy analysis and policy transfer theories were used to guide a deductive analysis for manifest and latent content.

**Results:**

According to stakeholders, the transition to the globally recommended child-appropriate dosage formulations has been slow in Uganda due to a number of factors. These factors include resource constraints at the global and national levels, lack of Ministry of Health (MOH) formal commitment to the adoption of the child-appropriate dosage formulations policy and a lack of consensus between those who advocated for the availability of liquid oral dosage formulations for easy administration and effectiveness and those who were more convinced by economic arguments and preferred the procurement of solid oral dosage formulations intended for adults.

**Conclusions:**

The global policy for child-appropriate dosage formulations still remains to be implemented in Uganda and other low income countries. This has been due to lack of resources that hindered formal transfer of the policy from the global to the local level. To achieve this transfer there is a need for resource mobilisation at both the international and local levels, together with the revitalisation of UMTAC to enable it to take on a leadership role of the coalitions supporting child-appropriate dosage formulations.

## Introduction

In 2007, the Sixtieth World Health Assembly (WHA) passed the resolution entitled “Better medicines for children”. The resolution urged Member States to include child-appropriate dosage formulations in their essential medicines lists to help improve access to better medicines for children and requested the World Health Organization (WHO) Director - General to develop the WHO Model List of Essential Medicines for Children and a Model Formulary for Children to accompany this list [1]. Since then WHO has been looking for funding to support these initiatives. The Bill and Melinda Gates Foundation funded a ‘Better medicines for children’ research programme in Ghana, India and Tanzania and this research built on the WHO-led “Make Medicines Child Size” initiative. A number of international organisations, industry researchers, health‐care providers, professional associations, academia, civil society and some governments endorsed the child-appropriate medicines initiative. As part of the initiative, WHO recommended the commissioning of independent studies on the development of paediatric medicines, their efficacy and safety. The WHO also pledged to provide better information on child specific medicines to prescribers, pharmacists and other health workers and to explore ways of fast-tracking the regulation of quality paediatric formulations [2]. However, child-appropriate dosage formulations have not been addressed in the Essential Medicines and Health Supplies List of Uganda (EMHSLU) 2012 [3] and are scarcely available in the peripheral public health facilities in Uganda [4].

For some time, liquid oral dosage formulations were being used for children below eight years as they were considered unable to swallow capsules and tablets. However, it was acknowledged that liquid oral dosage formulations were inappropriate for low income countries due to unfavourable climatic conditions and the challenges of handling and storage. Consequently the flexible solid oral dosage formulations and other paediatric medicines were given prominence to address the shortcomings of liquid oral dosage formulations [5].

In Sub-Saharan Africa, the challenges of appropriate formulations for children have been documented [6–8]. In Tanzania for example, key impediments to the accurate administration of medicines to children have included children vomiting crushed tablets that had been mixed with water [6]. In Uganda, uptake of praziquantel for schistosomiasis was found to be low among school children due to the unpalatable taste and the related side effects [7]. In Ethiopia, children died after being choked by de-worming tablets which were not specifically formulated for children [8]. These challenges identify the need for more child-appropriate dosage formulations especially for young children.

Hoebert and colleagues [9] argued that the policy making process is just as important as the policy document itself since the process must create a mechanism by which all stakeholders are brought together and a sense of collective ownership of the final policy achieved. The feasibility of adopting child-appropriate dosage formulations in low income countries such as Uganda will depend on the successful policy transfer from the global to the national level. A review of the status of policy provisions for child-appropriate medicines in Uganda highlighted the lack of reference to a child-appropriate medicine concept and inadequate provisions for child-appropriate formulations such as dispersible tablets-thereby indicating a limited attention or commitment to the WHO recommendations [3]. In Uganda, some stakeholders participated in the revision of the Essential Medicines and Health Supplies List of Uganda (EMHSLU) [10] and the Uganda Clinical Guidelines (UCG) in 2012 [11]. This study explored stakeholders’ views about the relevance of the global recommendation for child-appropriate dosage formulations in the context of Uganda.

## Methods

### Conceptual and theoretical approach

In this paper, we refer to the concept of child-appropriate dosage formulations that originated from the WHA resolution of ‘Better medicines for children’. Child-appropriate medicines refer to medicines that are available in formulations that allow doses to be easily adjusted to reflect the size, state of development, and condition of a child and can be easily administered. In the EMHSLU 2012, provisions for medicine according to age and weight of the child were addressed [3]. WHO recommended the dispersible, scored tablets or equivalent flexible solid oral dosage formulations as the most appropriate formulations [12, 13]. Other child-appropriate formulations include effervescent tablets, granules, sprinkles and non-oral formulations such as suppositories and injectables.

This study leans on theoretical discussions in the field of policy transfer, policy adoption and policy analysis. Dolowitz and Marsh [14] emphasised the need to contextualise the policy transfer processes, including the different actors involved, why the actors are involved and at what stages they get involved. They specify that policy transfers may occur due to different factors including global pressures, rapid growth in communication and the amount of information available to policy makers [14]. According to Evans [15], the relationship between systemic globalising forces and the increasing scope and intensity of policy transfer activity is crucial in determining policy adoption. For policy transfer from the global to the local level to be successful, cognitive barriers need to be addressed [15]. Walt and Gilson [16] in their policy analysis framework, focused on policy content, context, process and actors. In line with this, Sabatier and Jenkins, in their advocacy coalition framework, highlighted the importance of coalitions between actors who seek to influence government decisions and the inherent belief systems [17]. The aspect of belief systems was further accentuated by Shore and Wright [18] who highlighted the importance of values, interests and power relations in policy formulation and analysis.

### Study setting

This study was conducted in Uganda, one of the countries working closely with WHO to promote the rational medicines policy, supply, access and use. Uganda is a low income East African country with a population of around 35 million, according to the 2014 Population and Housing Census [19]. In 2012, the under-five mortality rate was 69 and the infant mortality rate 45 per 1000 live births [20].

In Uganda, children access medicines through public health facilities, private not for profit (PNFP) facilities, the private for profit sector and donor funded vertical programmes. Public and donor funded services are provided free of charge. The private sector provides about 50 % of the health services in the country [21]. International development partners have also been actively involved in medicines policy reforms and their implementation in Uganda since 2007. The main partners include UNICEF, WHO, Joint Medical Stores (JMS), the USAID funded project “Securing Ugandan’s Rights to Essential Medicines (SURE)”, STRIDES for family health and the Malaria Consortium (MC) project. In 2009, the government recentralised the procurement of medicines from the Districts to the National Medical Stores (NMS). The JMS complements NMS by procuring and distributing medicines for faith-based organisations (FBOs) representing Catholic and Protestant facilities. Both NMS and JMS work closely with the National Drug Authority (NDA) which controls production, importation and sales of medicines. NDA is responsible for the registration of new medicines and phasing out those deemed outdated.

### Study design

This was a qualitative study consisting of thirty four in-depth interviews with representatives of the stakeholder groups. In the context of this study, the word stakeholder refers to individuals and groups of people with an interest in medicines for children, including those who had been involved in the design and implementation of programmes, guidelines and setting priorities for public procurement and financing of medicines. The stakeholders included MOH officials, development partners, health workers and business (in medicines) community.

Twenty informants were purposively selected based on their professional involvement in relation to child health, pharmaceuticals, health policy and experience in child survival interventions. These included representatives from the key departments such as the Child Health Division and the Pharmacy Department of the Ministry of Health (MOH), National Medical Stores (NMS), Joint Medical Stores (JMS), National Drug Authority (NDA), private pharmacies, academic institutions and some development partners. The sample size was determined by data saturation for certain questions on the one hand and data gaps on the other. Fourteen additional respondents were identified and approached with guidance from the initial respondents where it was felt there were gaps in the information required. In-depth interviews were chosen to explore the informants’ knowledge, experiences, perceptions, attitudes and values about child- appropriate dosage formulations. Table 1 presents the list of informants.

**Table 1:** Categories of informants and their institutions

Category of respondents	Institution	Number
Ministry of Health	National Medical Stores	1
	National Drug Authority (NDA)	1
	Pharmacy	1
	Procurement	1
	Child Health	1
Donors and development partners	UNICEF Uganda	1
	UNICEF international	1
	WHO	1
	SURE	1
	STRIDES for Family Health	1
	Malaria Consortium	2
Professional bodies	Paediatricians	7
	Pharmacist Association	2
Private sector	Importers of medicines	2
	Retail pharmacists	6
	Local manufacturers	2
	Author of “The Precise Guide”	1
Private not for profit	Joint Medical Stores	1
Academia	Pharmacist	1

### Data collection

An interview guide was designed and used to collect data. The themes in the guide focused on concepts and elements of child-appropriate dosage formulations, respondents’ awareness of the concepts and their views about their feasibility in Uganda. Additionally stakeholders were asked about their opinions regarding child-appropriate formulations and the advantages and impediments to the adoption of child-appropriate formulations. The data collection guide was often supplemented with additional questions as and when necessary to probe for extra information. The first author with the help of a research assistant conducted the interviews in English, the official language of Uganda. The role of the research assistant was to take notes, record and transcribe the interview recordings. The research assistant is a university degree holder and experienced in qualitative data collection. The research assistant was thoroughly trained on the study objectives, the methodology and how to transcribe interviews.

Data collection was an iterative process and consequently additional questions were added as new, relevant issues emerged from some of the responses. Data gaps identified during the preliminary analysis were supplemented by conducting repeat interviews (two interviews) and recruiting new informants (10 informants) [22]. The interviews were digitally recorded if the respondent consented. The majority of the interviews were conducted in the respondents’ office/work place, whilst the rest were carried out outside the office for the convenience of the respondents. Each interview lasted between 45 and 60 min. A follow up workshop was held with stakeholders to present and validate the preliminary findings.

### Data analysis

Data analysis was conducted manually by the first author. A deductive content and thematic analysis was applied [23]. A deductive approach involves a theory guided analysis whereby themes adopted from the theoretical frameworks are then compared with the primary data. A coding sheet was developed, which included elements of the theoretical concepts of policy transfer, processes, role of actors, context, content, power, interests and values. The digitally recorded interviews and workshop proceedings were transcribed verbatim, printed and carefully read. The codes were then applied to the scripts.

### Ethical considerations

The study was approved by the School of Medicine Research and Ethics Committee of the College of Health Sciences, Makerere University and was cleared by the Uganda National Council of Science and Technology (Ref: SS 2703). On invitation, the informants were briefed about the purpose and content of the interview. Informants signed a consent form before the interviews commenced. The study findings were shared with stakeholders in a meeting that was organised by the research team. Policy briefs from the study were developed to share with the relevant stakeholders so as to initiate a dialogue aimed at making available child-appropriate formulations in the country.

## Results and discussion

The implementation of the globally recommended child-appropriate dosage formulations has been gradual in Uganda due to various factors that are also interrelated. The analysis revealed the following main themes:

- The failure of government to formally adopt the child-appropriate dosage formulations’ policy, primarily due to resource constraints;
- A lack of consensus between stakeholders who advocate for the availability of child-appropriate dosage formulations (liquid oral dosage formulations) and those who are more convinced by economic arguments and therefore prefer procuring only solid oral dosage formulations intended for adults and
- Leadership and coordination challenges.

### Government failure to formally adopt child-appropriate dosage formulations

There has been no official transfer of the child-appropriate medicine policy from the global to the local level in Uganda. At the global level, WHO and their partners developed the required documentation and member countries, perhaps with support from the WHO local offices, were to find resources, adopt and implement the policy. An informant who participated in the initial campaign for child-appropriate dosage formulations at the global level, noted that the official roll out of this campaign did not take place in most of the low-income countries due to the lack of resources.

*One of the challenges we are faced with is lack of resources to roll out the campaign worldwide. We have been waiting for donors to get on board and support the (make medicines child size) campaign but only Melinda Gates Foundation came up and only supported the pilot of the campaign in 3 countries: India, Ghana and Tanzania. We are still waiting for other donors, perhaps The Global Fund will come in and support (informant at the global level).*

None of our informants in Uganda had ever participated in a meeting that specifically focused on rolling out child-appropriate formulations. The WHO office in Kampala did not push for reforms towards child-appropriate dosage formulations, presumably knowing that the government could not afford these formulations at that time. Both the WHO local office in Kampala and MOH Uganda continued to anticipate global level initiatives such as the Global Fund to support the rollout of child-appropriate dosage formulations - a support that to date has not been realised.

*Although WHO’s position on this has been to advocate for child-appropriate dosage formulations rather than dividing up adults’ medicines, this position has not been so strong on that because we understand the economic and cost issues.… At the global level, WHO has been expecting support from the manufacturers. We at WHO do not fund the supply of medicines but can negotiate and lobby the Global Fund to provide funding (WHO Uganda official).*

The Ugandan MOH did not use the normative documents from WHO and their partners to initiate a medicine policy change dialogue. This, coupled with a rather hard-headed economic decision in favour of lower-priced adult dosage forms, paralysed both the confidence and authority to initiate policy in favour of child-appropriate dosage formulations. In this context, the MOH developed cold feet about the policy.

*The child size medicines concept which you have introduced may be a concept at the global stage that should be first developed there (at the global level) and later introduced as an affirmative action (special consideration) funded and spearheaded by the donor community (MOH official, child health division).*

Whereas some child-appropriate dosage formulations were available on the global market, poor countries like Uganda could not easily access them because they were initially too expensive and as highlighted in a recent study, the budget is already insufficient to procure all the essential medicines [24]. Although it is the responsibility of member states to adopt WHO proposals, the role of global influence in the initiation of the discussions and availability of resources cannot be underestimated. Another study in Uganda has demonstrated that changes in malaria treatment policies were possible due to WHO’s active involvement in the local discussions and a strengthened MOH financial capacity to lead the process [25].

It should be noted that the availability of resources is a critical factor in the consideration of policy analysis theories. It is largely because of costs that MOH officials prefer solid oral formulations for adults (to give to children) to the more evidence based, effective but expensive child-appropriate dosage formulations such as dispersible tablets. Despite being a vital criterion for selecting essential medicines, cost effectiveness was not a key consideration in the formulation of the medicine policies like elsewhere [26]. Similarly, the EMHSLU did not provide for child-appropriate dosage formulations such as dispersible tablets, granules and pellets [3]. This situation remains despite the WHA60 recommendation, requesting member states to “collaborate with governments, other organisations of the United Nations system, including WTO and WIPO, donor agencies, nongovernmental organisations and the pharmaceutical industry in order to encourage fair trade in safe and effective medicines for children and adequate financing for securing better access to medicines for children” [1].

Failure to officially roll out the policy from the global to the national level grossly affected the level of awareness that the stakeholders have about child-appropriate dosage formulations. It was evident in the study that stakeholders have varying levels of understanding of the concept of child-appropriate dosage formulations. Responses from the stakeholders reflected a range of diverse interpretations of what child-appropriate dosage formulations are supposed to be. Some associated them only with HIV/AIDS because at the time the HIV/AIDS Antiretroviral Therapy (ART) programme had introduced specific paediatric HIV management medicines. Some paediatricians simply generalised child-appropriate dosage formulations as syrups.

*These [child appropriate] are formulations for children. These are syrup formulations because children like sweet things we give them and besides they are easier to swallow (Paediatrician).*

One of the pharmacists, after some explanation, correctly associated the child-appropriate dosage formulation with familiar concepts of ‘paediatric formulations’ and ‘child-friendly medicines’.

***Interviewer:****Have you heard about child-appropriate dosage formulations?*

***Respondent****: No not really. Unless you elaborate a little more.*

***Interview:****The World Health Organization introduced the idea of increasing access to medicines that suit the child’s age and weight and which are easy to administer.*

***Respondent:****May be child-friendly and paediatric formulations, is that what you mean? (Pharmacist)*

Some of the paediatricians had no idea about the concepts.

***Interviewer:****what do you know about child-appropriate dosage formulations?*

***Respondent****: I beg your pardon*

***Interviewer****: These concepts of child size and child-appropriate dosage formulations, have you heard about them?*

***Respondent****: No, not really (Paediatrician-2)*

Although dispersible tablets are popular at the global level and were reported to be good for children by the Ugandan stakeholders, they were not considered to be feasible at the time because they were considered to be expensive. No respondent mentioned any other child-appropriate dosage formulations such as pellets, sprinkles or granules as child-appropriate dosage formulations. However, respondents regularly mentioned liquid oral dosage formulations or the dividing of solid oral dosage formulations for adults for children.

During the stakeholder meeting to validate the results of this study, participants demonstrated uncertainty about the concepts of ‘child size’ and ‘child-appropriate formulations’, how the campaign was rolled out from the global to the national level, who was targeted and the process that was applied. Participants also demonstrated a limited awareness of the child-appropriate dosage formulations such as the flexible oral dosage formulations. This limited understanding of the child-appropriate dosage formulations by key stakeholders was a barrier to their integration into the health policy. This confirms Evans [15] argument that, a globally designed policy (in this case the child-appropriate dosage formulations policy), can be adopted by a low income country (like Uganda) only if cognitive barriers and resource constraints at the global and national levels are addressed. Evans also emphasised the crucial role of the relationship between systemic globalising forces and the increasing scope and intensity of policy transfer activity in determining policy adoption and implementation [15].

There were contradictions between policy and practice that also affected the adoption of child-appropriate dosage formulations. One contradiction concerned the removal of liquid oral dosage formulations from the EMHSLU 2012 and health workers’ defiance of the policy by continuing to prescribe the unavailable liquid oral dosage formulations. Paediatricians referred the caregivers to procure the deleted (hence unavailable in public facilities) medicines from the private sector. Despite the fact that paediatricians preferred prescribing liquid oral dosage formulations for children, MOH officials on the other hand continued to procure solid oral dosage formulations for adults to be given to children

*The problem of adult medicines is very serious. We only get adult medicines and some caregivers also prefer syrups, they complain that the children vomit the tablets. I also think that syrups are better for very young children. Although the policy does not allow it, it is normal for us to prescribe the syrups so that caretakers can buy from the drug shops and pharmacies (paediatrician-3).*

During the revision of the essential medicines list, there was a heated debate about including liquid oral dosage formulations. The paediatricians were minority in the debate (as was reflected in the list of participants who drafted the medicine list) which was concluded with liquid oral dosage formulations being removed from the EMHSLU.

The other contradiction is that despite the absence of dispersible tablets in the EMHSLU, development partners have been distributing them in the community bypassing government health facilities. The Malaria Consortium, with support from UNICEF, has been working with Village Health Teams (VHTs) in 17 Districts distributing dispersible tablets of artemether-lumefantrine, amoxicillin, zinc sulphate and low osmolality oral rehydration salts (in powder form) as well as rectal artesunate for emergency use before referral in cases of severe fever in children. Because dispersible tablets were perceived to be more expensive, the MOH was reluctant to integrate some of the child-appropriate dosage formulations into the EMHSLU 2012 and scale-up their distribution countrywide. Although the MOH wanted a more balanced distribution covering both public health facilities and the community, they failed to act and continued to supply the conventional medicines in the public facilities.

*Partners using ICCM approach are attempting to bring these more expensive drugs nearer to the people by working through community based structures. We asked the partners to also distribute the same medicines in government facilities but we have not yet reached a compromise (MOH official child Health Division).*

This arrangement has created a “twin track” of child-appropriate dosage formulations in the community resulting in the non-child-appropriate dosage formulations (largely solid oral dosage formulations for adults) which are not evidence based being supplied by the government in public health facilities. This dual arrangement reflects the policy-practice gap, whereby donors, operating outside the policy framework have introduced child-appropriate dosage formulations and selected their own channels for distributing these medicines in the community. This dual track arrangement negates the referral function between the community and the formal healthcare system.

### Lack of consensus about liquid oral dosage formulations and solid oral dosage formulations intended for adults

Another bottleneck in the transition to child-appropriate dosage formulations was the lack of a common ground of agreement among stakeholders about formulations they thought were suitable for children as demonstrated in Table 2. There were those who advocated for the availability of oral liquids (the effectiveness view) and those who were more convinced by economic arguments and therefore preferred procuring only solid oral dosage formulations intended for adults (the efficiency view).

**Table 2:** Stakeholder Perspectives about child-appropriate medicines

Respondents	Perspectives and Preferences for Medicines for Children
MOH (child health division, pharmacy division and procurement)	Preference for solid oral dosage formulations for adults because they are cheaper than the liquid oral dosage formulations that are too expensive, bulky and have short shelf life.
MOH (National Medical Stores)	Supports the efficiency view. The choice of medicines to be procured should address wider public interest needs. Liquid oral formulations do not meet this requirement. Prefer solid oral dosage formulations for adults that are cheap and easy to handle.
MOH (National Drug Authority)	The mission of NDA is to ensure access to quality, safe and efficacious medicines through the regulation and control of their production, importation, distribution and use of the medicines. NDA has registered varieties of medicines including child-appropriate dosage formulations and liquid oral dosage formulations some of which are not included in the EMHSLU but are registered for the private sector.
Joint Medical Stores	Support for the effectiveness view. Liquid oral dosage formulations are most appropriate for administering to children. Dividing adult tablets leads to inaccurate doses and interference in chemical properties. Holds a view that the UCG are not consistent with EMHSLU.
Development Partners	Support the efficiency view since Uganda is a poor country that cannot afford liquid oral dosage formulations and dispersible tablets. Supported the removal of liquid oral dosage forms from the EMHSLU 2012.
Paediatricians	Support the effectiveness view and argue that liquid oral dosage formulations are easy to administer and are best for infants. Fear of adverse events that might arise due to inaccurate dosing when solid oral dosage formulations for adults are split. They have no trust in the UCG which do not adequately address children’s needs.
UNICEF	The child size medicine concept would be the ideal and if not adult medicines should be scored to make them easy to break for children. Supports distribution of child-appropriate medicines at the community level.
Pharmacists outside government	Dividing of adult medicines interferes with chemical properties which affects the effectiveness of the medicines and might lead to adverse events. Highlight the role the Trade-Related Intellectual Property Rights (TRIP) agreements which keep the medicine prices high and hence impossible for low income countries to purchase such medicines using their own budget.

The effectiveness view, mainly supported by paediatricians and some pharmacists, maintained that despite child-appropriate dosage formulations being expensive, they are still essential because they are the most effective form of treatment for children. They were opposed to the practice of breaking of solid oral dosage formulations for adults into quarters and halves, crushing tablets or opening capsules for children. Administering medicines in this way is difficult and can cause inaccurate dosing, which may result in reduced efficacy (due to under‐dosing) and/or safety (due to over‐dosing).

*Syrup gives you more precise and clearly calculated doses for the child. We need a lot of these syrup formulations especially for the very young. If you are going to get a tablet and start dividing it here and there you may not get the actual strengths of the drug that you want for a small child (Paediatrician-4).*

FBOs also supported the procurement of liquid oral dosage formulations because they could afford them using funds from their user fees and donor support. A representative of the FBOs argued that liquid oral dosage formulations were critical for children and that there was no way FBOs could discontinue their procurement.

Some of the pharmacists argued that splitting of adult tablets might destroy the surface properties of the medicines leading to problems of absorption. This happens when the tablet is “enteric coated” to prevent it from being absorbed before it reaches the desired segment in the gut (digestive system). Destruction of surface properties would undermine the effectiveness of the medicines with a likelihood of negative side effects.

*[….] a tablet which is for example enteric coated …. if you break it you are spoiling the properties of the coating […] if it is supposed to be digested in the stomach, breaking it means it is going to be digested immediately and will never give you the benefits of slow or delayed action (a pharmacist, representing a Civil Society Organization).*

The efficiency view was based on the understanding that Uganda, being a poor country, could not afford the more costly child-appropriate formulations. With this view in mind, the Ministry of Health programme managers, procurement institutions and development partners who financed the revision of the EMHSLU 2012 and the UCG 2010/2012 decided to support the exclusion of liquid oral dosage formulations on the grounds that they were expensive.

*We have removed most of the mixtures and syrups from the Essential Drug List because they are much more costly compared to tablets. And because we have so little money you have to choose between 2 children being treated with tablets only compared to one child getting a bottle of syrup. If I choose the 2 children and can’t afford the syrup, I can’t afford it. Because Uganda is a very poor country (Development Partner).*

*They (syrups) are very bulky. The cost of the syrup will also cover the bottle, the cup, packaging and the ingredients. Besides, that bottle can easily break. This poses storage problems. […] In the same space covered by one bottle for one child you can have tablets for more than 10 children. So you cut on the cost and also address things to do with storage. So it is much easy to handle tablets (Ministry of Health official, NMS).*

The MOH official’s argument against liquid oral dosage formulations concurs with the global concern about their unsuitability in low income settings. At the global level, alternatives to liquid oral dosage formulations recommended for paediatric patients include the flexible solid oral dosage formulations (e.g. dispersible tablets). The key decision makers, who hold the efficiency view, continue to emphasise the use of solid oral dosage formulations for adults to be given to children.

From the foregoing discussions, it is evident that liquid oral dosage formulations and solid oral dosage formulations for adults were defined as the most important formulations for children. This has diverted attention from the more child-appropriate formulations suitable for the low income conditions such as the dispersible tablets. Use of adult tablets to treat under five children is risky because studies worldwide have demonstrated that children have difficulties in swallowing tablets [6, 27–31]. Hill [31] recommended that replacing liquid oral dosage formulations with flexible solid oral dosage formulations (such as dispersible tablets), which could be given as liquids at the point of administration, would potentially be a huge advance in the provision of medicines for children. The question of evidence-based formulary decision-making is a problem not only in Uganda but also in other low-income countries. A study in Tanzania for example, showed that efficacy, safety, availability and affordability were the most frequently utilised criteria in decision-making, although these were largely based on experience and assumptions rather than evidence [26].

The context as described above indicates the challenges of providing viable pathways for the easy adoption of a child-appropriate dosage formulations policy in Uganda. This concurs with the theoretical arguments referred to earlier that underscored the importance of understanding the role of context, processes of transfer, values and interests [16, 18, 14]. Similarly, Orem and colleagues [32] argued that policy makers in Uganda might reject evidence if it is not in their favour or because of political interests and economic viability. A similar study of pharmaceutical reforms in Tanzania also showed that policy reforms were based on vested interests as opposed to the efficiency and effectiveness of the regulations [33]. Interests in cheaper adult formulations in the case of Uganda overwhelm those of evidence based child-appropriate dosage formulations that are child friendly, safer and more effective.

### Leadership and coordination challenges

There are gaps in terms of leadership and coordination of the discussions needed to develop a road map for child-appropriate dosage formulations in Uganda. The limited progress towards adoption of child-appropriate dosage formulations is affected by the weak institutional capacity to bring together key stakeholders to discuss the global recommendations. The Uganda Medicines and Therapeutic Committee (UMTAC) is responsible for drafting medicine policy reforms and is not sufficiently endowed with the financial resources to organise the discussions that would advocate for the integration of child-appropriate dosage formulations. For example, UMTAC has not been sitting regularly because of the failure to pay sitting allowances for the committee members. According to personal communication with one of the ministry officials, most of the successful workshops led by UMTAC (such as the revision of essential medicines lists and clinical guidelines) were donor funded. An additional problem is the lack of engagement with UMTAC from the technical departments whose responsibility it is to initiate and concretise the policy and there is rarely any feedback on the drafts of policy documents. According to Stone [34], a key feature of a policy network is a shared problem on which there is an exchange of information, debate, disagreement, persuasion and a search for solutions and policy responses. Sabatier and Jenkins [17] emphasised the need for coalitions between actors to influence government decisions and the inherent belief systems. Coalitions will work if there are strong institutional mechanisms to mobilise resources and coordinate coalition activities. In Uganda, coalitions to support child-appropriate dosage formulations are yet to be realised despite the urgent need for such coalitions.

### Strengths and limitations

Preliminary findings of the current study were disseminated in several workshops at the national and district levels. These have sparked off discussions about the ‘child size medicines’ concept ministerial and partner level. For example, new studies have been initiated to roll out and scale up pre-packaged dispersible tablets of amoxicillin for children in the communities and more recently, how this could be scaled up in the entire country including the public health facilities.

This was a qualitative study using in-depth interviews that sought to establish the awareness, knowledge and perceptions of stakeholders about the relevancy of ‘child-appropriate’ formulations in Uganda and to solicit for substantial information about the stakeholders’ awareness and perceptions about the reality of child-appropriate formulations in Uganda. Theory was a key consideration in the study design, data collection, analysis and interpretation. Several authorities in policy analysis have alluded to the importance of theory in policy analysis [16, 35, 36].

To ensure validity and reliability, informants were selected based on their professional experience and relevance to child health medicine policy reforms, pharmaceutical policies, and health policy experience and child survival interventions. To ensure consistency of the information obtained, an interview guide was used for all the interviews which were conducted by the first author who had designed the study, understood the study questions and maintained the same level of in-depth interviewing and probing for the key areas of interest throughout the interviews. This was done to maximise reliability, consistency and validity of the information obtained. The research assistant was thoroughly trained on study rationale, how to take notes and how to transcribe the data. The first author went through all the transcripts to acquaint himself with the data and check the quality of the transcripts. An iterative process was applied during data collection whereby issues that emerged in the initial interviews were followed up and confirmed in the subsequent interviews. In this way we were able to identify areas of convergence or divergence between stakeholders, their areas of interest and barriers that hindered easy adoption of child-appropriate medicine formulations. Where it was possible, more than one informant was recruited from the institutions to check consistency of the information obtained.

A similar approach of a deductive analysis using manifest and latent content can be used in similar studies in other low income countries. It would be useful for such studies to borrow from the theoretical constructs of policy transfer and formalisation processes, resource availability, institutional interests and their capacity to advocate for adoption of child-appropriate dosage formulations.

This study discusses a topical subject and contributes to the debate about the efforts aimed at reducing under-five mortality at the international and local levels. For these goals to be achieved, access to efficacious, evidence based, safe and child-appropriate dosage formulations are essential. In this study, some of the barriers to the realisation of the recommended child-appropriate dosage formulations have been identified and can be addressed by those with a mandate.

The study focused on the national level stakeholders who contribute to policy and may not necessarily implement them. Therefore, it was not possible to determine the status of child-appropriate formulations at the implementation level as there is often a divergence between policy and practice. Studies about availability of ‘child size medicines’ in the public health facilities, health workers’ experiences and caretaker preferences are needed to address this gap. Some stakeholders such as political representatives, implementers and users of the policy such as health workers and caretakers were not interviewed. We strongly believe that they are key stakeholders who should be included in subsequent studies.

## Conclusions and recommendations

This study has demonstrated that the slow transition to child-appropriate medicine formulation in Uganda has been due to resource constraints, lack of sufficient influence of the medicine policy coordinating institutions and a lack of common ground, divergent interests and values among the stakeholders. Countries in a similar situation to Uganda with an interest in adopting the child-appropriate medicines formulation policy will need to establish a platform to resolve professional, institutional and economic barriers to ensure successful policy outcomes. The globally designed child-appropriate formulations are still absent in the low income countries for which they were designed due to financial limitations and gaps in policy transfer and adoption. Therefore low income countries like Uganda should formally adopt the policy. This would require revising the EMHSLU and UCG to include child-appropriate dosage formulations. The needed policy reforms will be achieved through the mobilisation of resources and by establishing coalitions of interest groups to advocate and lobby for support of the policy. This will need to include campaigns both to promote the uptake of available, evidence based paediatric formulations whilst also working to encourage the pharmaceutical companies capacity and willingness to formulate medicines for children [2]. WHO and their partners should consider working with other international organisations, pharmaceutical companies and other donors to do ‘a negotiated’ policy transfer in favour of child-appropriate formulations such as the integration of dispersible tablets into the essential medicine lists of low income countries.

## References

[1] World Health Assembly; Resolution WHA 60.20 2007Better medicines for children,. http://apps.who.int/medicinedocs/en/d/Js21455ar/. Accessed 19 March 2015.

[2] Finney E. Children’s medicines: a situation analysis November 2011. Retrieved from: Make medicines child size; http://apps.who.int/medicinedocs/en/d/Js20020en/. Date accessed 06/13/2015

[3] Nsabagasani X, Ogwal-Okeng J, Mbonye A, Ssengooba F, Nantanda R, Muyinda H, Holme Hansen E (2015). The “child size medicines” concept: policy provisions in Uganda. J Pharm Policy Pract.

[4] Nsabagasani X, Ogwal-Okeng J, Mbonye A, Ssengooba F, Muhumuza S, Hansen EH (2015). Availability and utilization of the WHO recommended priority lifesaving medicines for under five-year old children in public health. J Pharm Policy Pract.

[5] Stoltenberg I, Breitkreutz J (2011). Orally disintegrating mini-tablets (ODMTs)--a novel solid oral dosage form for paediatric use. Eur J Pharmac Biopharm.

[6] Adams LV, Craig SR, Mmbaga EJ, Naburi H, Lahey T, Nutt CT, Kisenge R, Noel GJ, Spielberg SP (2013). Children’s Medicines in Tanzania: A National Survey of Administration Practices and Preferences. PLoS One.

[7] Muhumuza S, Olsen A, Katahoire A, Nuwaha F (2013). Uptake of preventive treatment for intestinal schistosomiasis among school children in Jinja district, Uganda: a cross sectional study. PLoS One.

[8] Keiser J, Ingram K, Utzinger J (2011). Antiparasitic drugs for paediatrics: systematic review, formulations, pharmacokinetics, safety, efficacy and implications for control. Parasitology.

[9] Hoebert JM, van Dijk L, Mantel-Teeuwisse AK, Leufkens HG, Laing RO (2013). National medicines policies - a review of the evolution and development processes. J Pharm Policy Pract.

[10] Government of Uganda (2012). Essential Medicines and Health Supplies List for Uganda 2012 (EMHSLU).

[11] Government of Uganda. Uganda Clinical Guidelines (2010). National Guidelines on Management of Common Conditions.

[12] World Health Organization. Priority medicines for mothers and children. Geneva: WHO; 2011. http://cdrwww.who.int/medicines/publications/A3prioritymedicines.pdf. Accessed 19 March 2015.

[13] World Health Organization. Priority life-saving medicines for women and children. WHO; 2012. http://www.who.int/reproductivehealth/publications/general/emp_mar2012.1/en/ Accessed 19 March 2015.

[14] Dolowitz DP, Marsh D (2000). Learning from abroad: The Role of Policy Transfer in the Contemporary Policy Making. Gov: Inter J Policy Adm..

[15] Evans M (2009). Policy transfer in critical perspective. Policy studies.

[16] Walt G, Gilson L (1994). Reforming the health sector in developing countries: the central role of policy analysis. Health Policy Plan.

[17] Sabatier PA, Jenkins-Smith HC (1988). Policy Change and Learning: An Advocacy Coalition Approach.

[18] Shore C, Wright S (1997). Policy: A new field of anthropology.

[19] Republic of Uganda (2014). National Population and Housing Census: Provisional Results.

[20] UNICEF. Uganda-Unicef Statistics In*.*: UNICEF; 2010.

[21] Government of Uganda (2010). Health Sector Strategic and Investment Plan: Promoting People’s Health to Enhance Socio-Economic Development.

[22] Patton MQ (2002). Qualitative Research and Evaluation Methods.

[23] Graneheim U, Lundman B (2004). Qualitative content analysis in nursing research: concepts, procedures and measures to achieve trustworthiness. Nurse Educ Today.

[24] Ministry of Health Uganda, SURE Program (2014). Improving equity in resource allocation for essential medicnes and health supplies in public sector health facilities in Uganda.

[25] Nabyonga-Orem J, Ssengooba F, Macq J, Criel B (2014). Malaria treatment policy change in Uganda: what role did evidence play?. Malar J.

[26] Mori AT, Kaale EA, Ngalesoni F, Norheim OF, Robberstad B (2014). The Role of Evidence in the Decision-Making Process of Selecting Essential Medicines in Developing Countries: The Case of Tanzania. PLoS One.

[27] Hansen DL, Tulinius D, Hansen EH (2008). Adolescents’ struggles with swallowing tablets: barriers, strategies and learning. Pharm World Sci.

[28] Gitanjali B (2011). Essential medicines for children: Should we focus on a priority list of medicines for the present?. J Pharmacol Pharmacotherapeutics.

[29] Quinzler R, Gasse C, Schneider A, Kaufmann-Kolle P, Szecsenyi J, Haefeli WE (2006). The frequency of inappropriate tablet splitting in primary care. Eur J Clin Pharmacol.

[30] Nunn T, Williams J (2005). Formulation of medicines for children. Br J Clin Pharmacol.

[31] Hill SR (2012). Putting the priorities first: medicines for maternal and child health. Bull World Health Organ.

[32] Orem JN, Mafigiri DK, Marchal B, Ssengooba F, Macq J, Criel B (2012). Research, evidence and policymaking: the perspectives of policy actors on improving uptake of evidence in health policy development and implementation in Uganda. BMC Public Health.

[33] Mori AT, Kaale EA, Risha P (2013). Reforms: a quest for efficiency or an opportunity for vested interests’? A case study of pharmaceutical policy reforms in Tanzania. BMC Public Health.

[34] Stone D (2012). Transfer and translation of policy. Policy studies.

[35] Walt G, Shiffman J, Schneider H, Murray SF, Brugha R, Gilson L (2008). ‘Doing’ health policy analysis: methodological and conceptual reflections and challenges. Health Policy Plan.

[36] Stephen H (2001). Policy Analysis.

